# An in silico epitope-based peptide vaccine design against the 2019-nCoV

**DOI:** 10.1186/s43042-020-00071-7

**Published:** 2020-07-27

**Authors:** Olanrewaju Ayodeji Durojaye, Talifhani Mushiana, Samuel Cosmas, Glory Omini Ibiang, Mercy Omini Ibiang

**Affiliations:** 1 School of Life Sciences, Department of Molecular and Cell Biology, University of Science and Technology of China, Hefei, China; 2 School of Chemistry and Material Sciences, Department of Chemistry, University of Science and Technology of China, Hefei, China; 3 Department of Biochemistry, University of Nigeria, Nsukka, Enugu State Nigeria; 4 Department of Biological Sciences, Coal City University, Emene, Enugu State Nigeria

Dear Editor,

The 2019-nCoV is a novel SARS coronavirus which was first isolated from three individuals having pneumonia with connection to the Wuhan epidemic of the severe respiratory illness [1]. The 2019-nCoV shares a close relationship with the original SARS-CoV, and it is believed to exhibit a zoonotic property. Genomic analysis of the virus has shown that it clusters genetically with the *Beta coronavirus* genus, alongside two other strains derived from bat. It shares a 96% identity with other bat coronavirus samples (Bat Cov RaTG 13) at the whole genome level. Chinese researchers in February 2020 discovered the amino acid difference in specific parts of the human and pangolin virus genome sequences, however, whole-genome comparison between the pangolin coronavirus, and the 2019-nCoV found a maximum of 92% identical genetic materials, which has so far not been sufficient enough to confirm pangolins to be the viral intermediate host [2].

Vaccines have been produced to target several animal coronavirus diseases, which includes the canine coronavirus, the infectious bronchitis virus of birds, and feline coronavirus. Previous efforts aimed at the development of antiviral vaccines for the *Coronaviridae* family that majorly affects humans that have been targeted at the Middle East respiratory syndrome and severe acute respiratory syndrome coronavirus. The MERS and SARS vaccines have been tried in animal models and up till February 2020, there has been no cure or protective vaccine that has exhibited safety and efficacy in humans [3].

The historical immunotherapy consensus has been about the targeting of easily accessible antibody-binding extracellular antigens only. The reason for this is because the antibodies which are of higher molecular weight stop the antigens from gaining access to their intracellular targets through the crossing of the cell membrane. In consistence with this thought train, approved therapeutic antibody targets are mostly extracellular antigens [4]. Three broad approaches more recently have been used in intracellular antigen targeting. It is not impossible for normally intracellular antigens that become externalized to be targeted by antibodies or their derivatives in a disease state. It is also not impossible to engineer cell-penetrating antibodies or fragments of antibodies and even antibodies whose expression is intracellular, with the aid of gene therapy. Finally, cell surface MHC-I-binding antibodies can be generated (major histocompatibility complex class I) [5].

With reference to previous virus related in-silico vaccine design studies [6, 7], we designed a new potential vaccine candidate using the main proteinase of the 2019-nCoV as the target protein. The viral main proteinase coding sequence was mapped out from its full genome which has been made accessible for the public in the database of Genbank (https://www.ncbi.nlm.nih.gov/nuccore/MN908947.3?report=fasta) with the accession number “MN908947.3” (Additional file 1). The sequence which ranges from the 10055 to 10972 nucleotides of the viral genome was translated, and the amino acid sequence was used in the 3D structural homology protein model prediction. A total of 120 templates were found, and an initial HHblits profile was designed by making use of the outlined procedure in Remmert et al. [8]. In the vaccine development process, we engaged the BCEPred which predicts the antigenic region of proteins based on individual or combination of different physico-chemical properties (flexibility/mobility, polarity, hydrophilicity, turns, accessibility, and exposed surface). Observations has been made as regarding the combination of these properties which showed that combining two or more confers a better accuracy when compared to a single property. Previous studies have revealed that the combination of the flexibility, hydrophilicity, exposed surface, and polarity properties of proteins produces a better performance on comparison to any other combination at a 2.38 threshold [9]. We therefore selected these properties in our B-cell epitope prediction process. The resulting peptide with the best epitope properties is a sequence of 15 amino acids (92-DTANPKTPKYKFVRI-106) which gave the highest epitope value of 3.053 (Fig. 1).

**Fig. 1:**
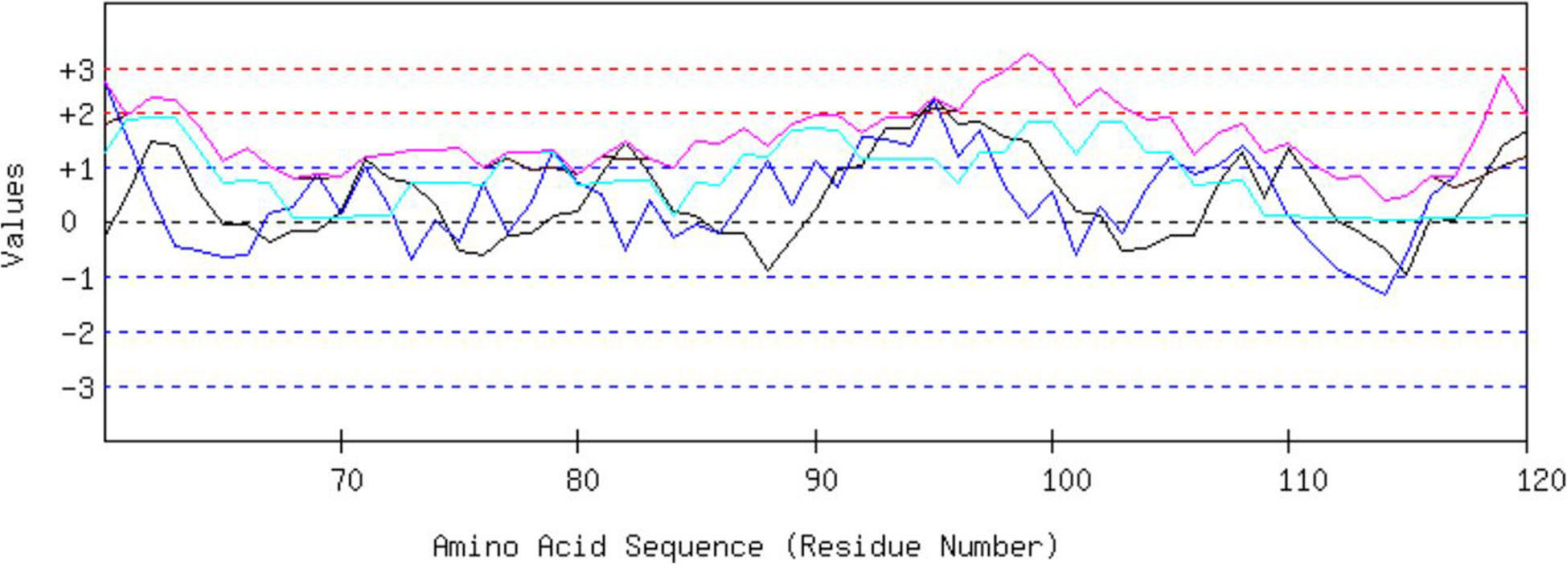
The graphical output format of the prediction of B-cell epitope by BCPred, which is a plot of the epitope values against the residue number. The graph uses a scale which is normalized between + 3 and − 3, with high values giving rise to the peaks. The different colors of the peak lines denote the individual physiochemical properties in which the prediction was based on. The blue, black, cyan, and purple colored peak lines as shown in the figure denote the flexibility, hydrophilicity, polarity, and combined physiochemical properties respectively

We went further to confirm the potential of the predicted B-cell epitope in generating high affinity antibodies through T-cell epitope prediction. This was achieved using the SYFPEITHI prediction server (database for MHC ligands and peptide motifs) [10]. This tool gives room for the detection of the ligation strength to a defined HLA type for a sequence of amino acids. The algorithms used are based on the book “MHC Ligands and Peptide Motifs.” The probability of being processed and presented is given in order to predict T-cell epitopes. The predicted T-cell epitope with the highest score is a nonamer which covers the second amino acid to the tenth (TANPKTPKY). This prediction was validated using the IEDB analysis resource consensus tool, which is another T-cell epitope prediction server [11]. The HLA class II binding regions of the antigenic sequence were predicted using the HLAPred server [12], which allows the identification of peptides that can bind with both the HLA class I and class II from the antigenic sequence. The HLAPred output shows the HLA class II prediction according to four selected alleles in an HTML mapping display format (Fig. 2). The 104-VRI-106 segment of the B-cell epitope was predicted to be a promiscuous binder as shown in Fig. 2. The promiscuous binding regions are those which bind with many HLA alleles.

**Fig. 2:**
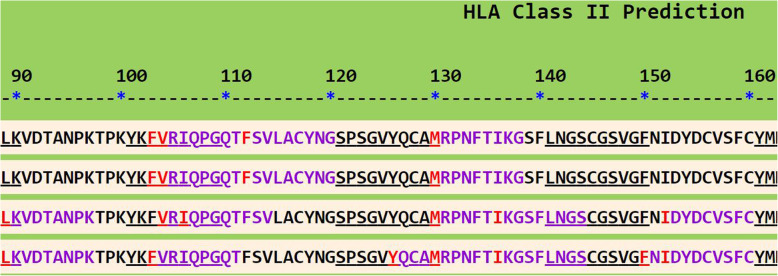
Depicts the HLA class II binding regions of the antigenic sequence with focus on the predicted segment of the B-cell epitope. The four selected alleles are HLA-DRB1*0101, 0102, 0301 and 0305, from top to bottom respectively. The N-terminals of predicted binders are shown in red and all other residues in blue color

Viral internalization greatly depends on glycosylation sites present on the viral protein. *N*-glycosylation sites on the 2019-nCoV main proteinase were therefore predicted using the NetNGlyc 1.0 prediction tool (Fig. 3) [13]. The graph illustrates predicted N-glycosylation sites across the protein chain where the x-axis represents protein length from the amino terminal to the carboxyl terminal. The position with a potential (the green vertical lines) crossing the threshold (red horizontal line) is predicted glycosylated.

**Fig. 3:**
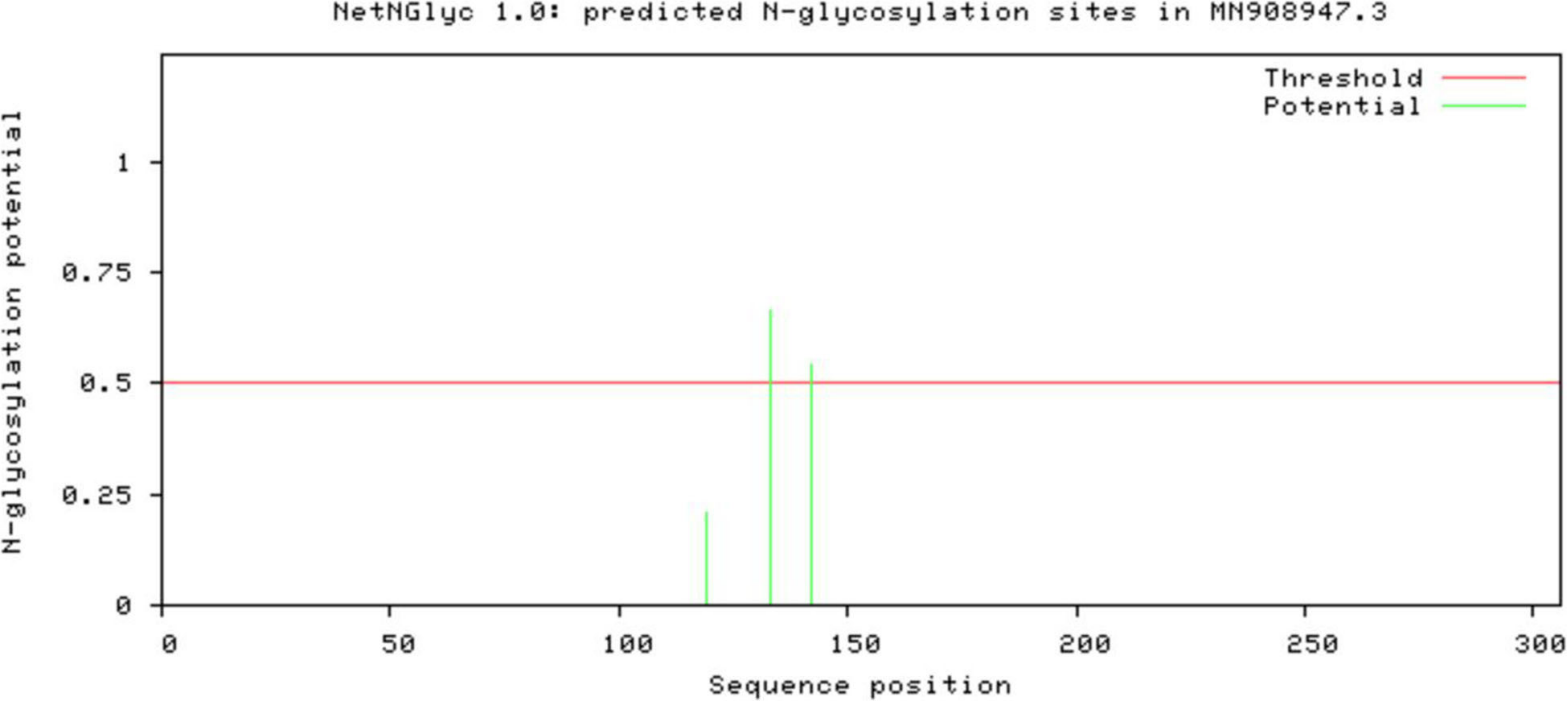
Graphical output of the predicted *N*-glycosylation sites in the viral main proteinase amino acid sequence. The output shows a plot of the *N*-glycosylation potential against sequence position

Sequences and structural motifs in polypeptide chains that classified and determined by comparative analysis make up the protein’s conserved domain. These domains are used in molecular evolution as building blocks which may undergo different forms of arrangements and recombination to produce proteins with varying functions. The importance of the conserved domains as evolutionary elements has led us into determining the level of conservation of the 2019-nCoV main proteinase epitope region. This was achieved using the conserved domain database (CDD) [14]. The conserved region of the protein covers the 29th amino acid of the sequence to the last, with the inclusion of the predicted epitope sequence (Fig. 4).

**Fig. 4:**
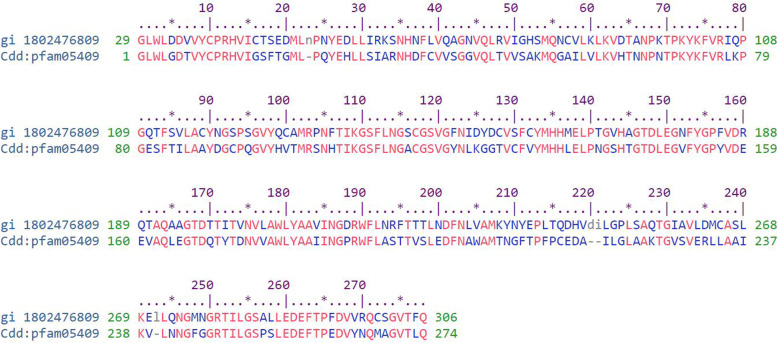
Conserved domain alignment output. The coronavirus endopeptidase C30 which corresponds to the Merops family C30. These peptidases are involved in viral polyprotein processing in replication and are conserved in the protein family

The physiochemical properties of the final peptide as predicted by the Expasy ProtParam server [15] predicted a molecular weight of 1778.08 Da with a theoretical *pI* of 10 indicating an alkaline protein. The half-life assessment was predicted to be 30 h in vitro in mammalian reticulocytes, > 20 h in yeast, and > 10 h in vivo in *E. coli*. The aliphatic index estimation predicted a score of 52.00, which indicates thermostability. The predicted GRAVY score was − 0.407, indicating a hydrophilic protein which is consistent with the 3D structural view of the protein (Fig. 5).

**Fig. 5:**
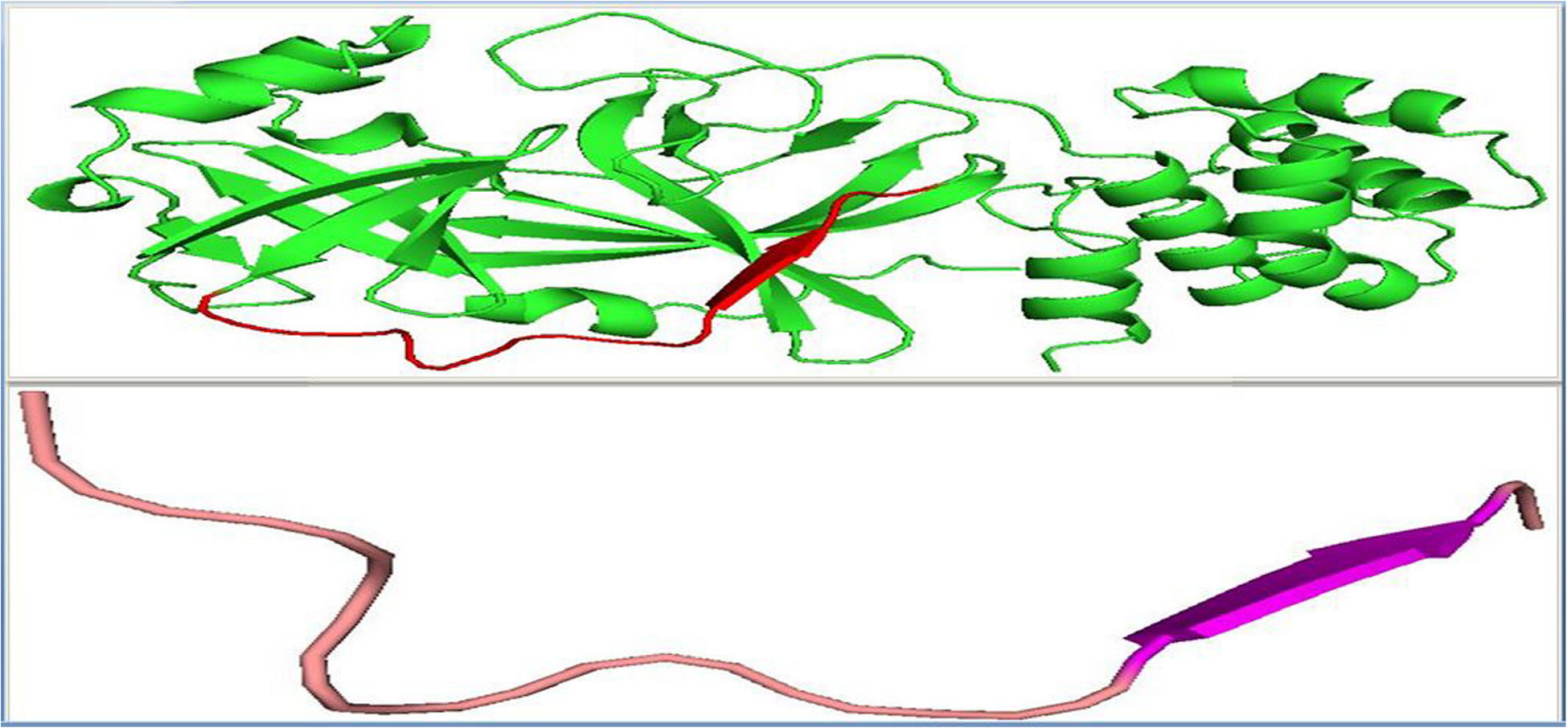
The first column is a 3D view of the 2019-nCoV main proteinase with highlighted antigenic region (red) while the second column shows the loop dominated secondary structure of the antigenic peptide

The secondary structures of the protein antigenic region as viewed in the Pymol molecular visualizer showed a loop dominated peptide with no helices (Fig. 5). We hereby recommend this peptide for further in vitro and in vivo studies as our in silico study that has predicted this region of the 2019-nCoV main proteinase as a potential B-cell epitope for a potent vaccine design against the virus.

## Supplementary information

**Additional file 1.**http://www.syfpeithi.de/bin/MHCServer.dll/EpitopePrediction?Motif=HLA-A*01&amers=9&SEQU=DTANPKTPKYKFVRI&DoIT=++Run++, https://www.ncbi.nlm.nih.gov/Structure/cdd/wrpsb.cgi.

## Data Availability

URL links of supplementary files are available in Additional file 1.

## Abbreviations

2019-nCoV: 2019 novel coronavirus
SARS-CoV: Severe acute respiratory syndrome coronavirus
MERS: Middle East respiratory syndrome
SARS: Severe acute respiratory syndrome
MHC: Major histocompatibility complex
HLA: Human leukocyte antigen
*pI*: Isoelectric point
